# Nutritional Supplementation of Yogurt with Jerusalem Artichoke Tubers: Organic Acid Profiles and Quality Parameters

**DOI:** 10.3390/plants11223086

**Published:** 2022-11-14

**Authors:** Ashwell R. Ndhlala, Arzu Kavaz Yüksel, Mehmet Yüksel

**Affiliations:** 1 Green Biotechnologies Research Centre, School of Agricultural and Environmental Sciences, University of Limpopo, Private Bag X1106, Sovenga 0727, South Africa; 2 Department of Food Technology, Technical Sciences Vocational School, Atatürk University, Erzurum 25030, Türkiye; 3 Department of Food Engineering, Faculty of Agriculture, Atatürk University, Erzurum 25030, Türkiye

**Keywords:** (*Helianthus tuberosus* L.) Jerusalem artichoke, yogurt, physicochemical, sensory, organic acid

## Abstract

Jerusalem artichoke (*Helianthus tuberosus* L.), also called wild sunflower, belongs to the Asteraceae family and is cultivated widely across the temperate zone for its nutritious tuber, which is used as a root vegetable. In this study, the Jerusalem artichoke (JA) was used as a supplementary additive for producing a functional yogurt, with enhanced health benefits and improving the microbiological, rheological, and sensorial quality characteristics of the product. The effects of the three different concentrations (1%, 2%, and 3%, *w*/*w*) of JA on the physicochemical properties, bacterial counts, sensorial properties, and organic acid profiles of yogurts were determined after 1, 7, 14, and 21 days of storage at ±4 °C. The results obtained revealed that with the addition of different concentrations of JA the overall parameters were statistically significant (*p* < 0.01 and *p* < 0.05) except for apparent viscosity, *Streptococcus thermophilus,* yeast and mold count, pyruvic ratios, and scores of flavor. Similarly, some parameters (fat ratio, yeast and mold count, concentrations of propionic, acetic, pyruvic, orotic, and lactic acids, and scores of appearance, consistency, and odor) changed depending on the storage time, while some did not show any changes regarding storage time. There was a relationship found between the concentration of JA and organic acid ratio (except for pyruvic acid) concentration in the yogurt samples. In conclusion, the research revealed the effect of JA in yogurt production as a thickener, flavor enhancer, prebiotic agent, and source of organic acids and bioactive compounds. The results indicate that JA has a good potential for enhancing the nutritional and physicochemical properties of the studied yogurt.

## 1. Introduction

**Figure 1:**
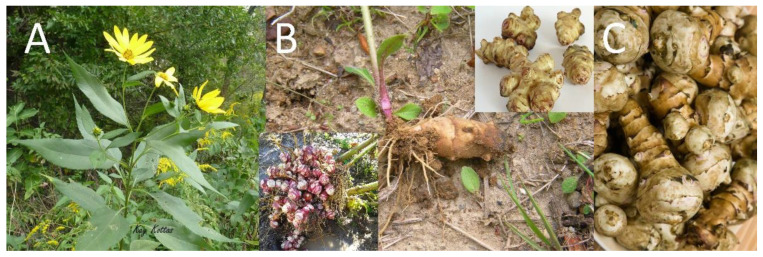
Jerusalem artichoke (*Helianthus tuberosus* L.): (**A**) the whole plant, leaves, and flowers, (**B**) the rhizome system, and (**C**) tubers.

Jerusalem artichoke (*Helianthus tuberosus* L.) belongs to the Asteraceae family and shows genetic variability in terms of genotypes. It grows naturally in the North American plains and is cultivated in many different parts of the world. The Jerusalem artichoke (JA) is a plant of high importance for human and animal nutrition and health [1,2,3]. The plant tuber has both functional (medicinal) and nutritional properties, and is especially beneficial for obesity and type 2 diabetes [4]. JA is an annual plant that is composed of a stem approximately 1–3 m tall, hairy oval leaves, yellow flowers, and a rhizome system that has small tubers (Figure 1) [1,5,6,7]. Its rapid growth causes natural control against weeds. JA is an advantageous traditionally cultivated crop due to it tolerance to diseases, tolerance to poor soil conditions, high-growth rate, and close to zero fertilizer requirements with a high resistance in terms of preventing plant diseases [8].

JA contains essential amino acids, proteins, minerals, and bioactive and functional components such as oligofructose, inulin, fructose, and flavonoids [9]. Because of its quality characteristics and prebiotic properties, JA is valued in the cosmetics, pharmaceutical, food, feed, sugar, paper, and bioethanol industries [10]. An important property of JA is that its tubers have inulin, naturally. Inulin is the main sugar in JA and is used by microorganisms for the production of fructose syrup, dietary fibers, bioethanol, and other biochemical materials [11,12]. Conventionally, JA has been evaluated for animal feed or food, and science for two decades, and used alternatively for the production of functional food ingredients [13,14,15]. Additionally, an analysis of literature sources revealed that Jerusalem artichoke is a multifunctional agricultural crop and can serve as a raw material for the production of some functional and dietary products, biologically active compounds, and food supplements. Tubers are used in the production of organic acids [1,16].

In recent years, inulin is the most commonly considered sugar in yogurt production in terms of prebiotic properties. Inulin consists of fructooligosaccharides and shows indigestible carbohydrate properties [17,18]. Inulin is a functional prebiotic agent suitable for the treatment of obesity, type 2 diabetes, and other blood sugar-related disorders that cannot be digested by the human gastrointestinal tract [4,19].

Yogurt is one of the most liked and consumed fermented milk products in the world [20]. It is fermented with lactic acid fermentation by *Lactobacillus bulgaricus* and *Streptococcus thermophilus* and has high-functional properties [21]. The flavor of yogurt is caused by the metabolism of the specific yogurt bacteria [22,23]. Prebiotics stimulate the activity of probiotics and yogurt starter cultures in the yogurt and human intestinal system. Inulin is a natural and indigestible carbohydrate that consists of fructooligosaccharides obtained from some fruits and vegetables and JA is one of its natural sources [17,18].

Organic acids show an effective role in terms of sensory and protective properties in yogurt production. Important organic acids are available in yogurt as a result of the growth of lactic acid bacteria including pyruvic acid, acetic acid, lactic acid, formic acid, propionic acid (carbohydrate metabolism), butyric acid (fat metabolism), citric acid, malic acid, etc., (from fruit and vegetables) [24,25]. Organic acids affect the flavor, quality, acidity, sensory characteristics, and microbiological quality of types of fermented milk. Furthermore, organic acids prevent the growth of unwanted microorganisms causing the spoilage of milk and dairy products [25,26].

JA had been known for its nutritional value and health-promoting effects in terms of its rich fiber, natural sugars, phytochemical contents, protein, and other water-soluble substances [16]. Numerous investigations have shown that JA tubers can be used as bioactive ingredients in dairy products (yogurt and cheese), bakery products (cake, biscuits, and bread), sausages, and beverages [27,28,29,30]. There are a lot of studies about yogurt with added JA in the literature. However, there have not been any studies about the JA addition on different properties and organic acid profiles of yogurt. The objective of this study is to determine the physical and chemical properties, microbiological counts, organic acid profiles, and sensory properties of yogurts produced with three different JA concentrations and kept for different storage periods.

## 2. Results and Discussion

### 2.1. Physicochemical Properties of JA-Supplemented Yogurt Samples

The changing physicochemical characteristics of the yogurt samples are given in Table 1. Total solids, ash, and protein values showed a similar trend in terms of the supplementation with different concentrations of JA. The differences among the yogurt samples were found to be statistically significant (*p* < 0.05; *p* < 0.01), while the storage periods did not show an effect on these parameters (Table 1). When the fat results were observed, it was determined that the first three samples were similar, while the JA_3_% sample was different from the others at *p* < 0.01. Yogurt fat content was reduced depending on the increase in the concentration of JA. Since the fat content of the JA is low, it caused a proportional decrease in the yogurt samples.

The highest viscosity value (7400 ± 423.42 cP) was measured in JA_3%_ and the lowest mean value (6950 ± 504.27 cP) was observed in the control. All of the samples and storage periods had similar trends in terms of statistical evaluations (Table 1). Generally, the viscosity parameters of yogurt samples exhibited an increment with the enhancement of the JA concentration and storage time. This may be due to the increase in the total solids content, fiber, oligosaccharides (especially inulin), and water holding capacity [31,32]. Additionally, inulin from the JA, as an amphiphilic molecule, can form many hydrogen bonds with protein molecules and increases the networking of gel [33]. All yogurts supplemented with JA exhibited viscoelastic behaviour.

Serum separation is defined as spontaneous water release from the milk gel. As a result of this situation, water accumulation occurs on the surface of the yogurt [34]. As presented in Table 1, the highest mean value (5.85 ± 0.37 mL/ 25 g) of syneresis value was obtained from the control and the lowest mean value (4.62 ± 0.40 mL/25 g) was exhibited by the JA_3%_. Statistical evaluations demonstrated that JA_2%_ and JA_3%_ samples showed similar trends to each other, while other samples were different from each other, and these samples were statistically significant (*p* < 0.01). The syneresis ratio of the yogurt samples was not affected by the storage period, as interpreted statistically. The results also showed that inulin, found in JA, had an effect on serum separation prevention [35]. The results obtained here might have stemmed from the increasing concentration of JA. So, water-holding capacity increased with due to the oligosaccharides, especially inulin from the tubers. Kavaz, Yüksel and Bakırcı [36] reported that inulin showed high water-binding properties. The observed results might also be due to JA’s fibrous structure and inulin ratio. Moreover, JA has a complex biopolymer contained of nonionic and ionic molecules at the appropriate pH values. A similar result was obtained by Guven et al. [37] and Bakr et al. [32].

As presented in Table 1, titratable acidity values (%) of yogurt samples increased with the increment of JA concentration, while the pH values showed a decrease. This decrease in pH values with increasing JA addition could be attributed to increasing the inulin content of treatments promoted by the growth of cultured microorganisms and the fermentation of lactose into lactic acid [38]. Statistical evaluations showed differences in titration acidity (*p* < 0.01) and pH (*p* < 0.05) with respect to the addition of JA. However, titration acidity (%) and pH values did not show a statistical change with different storage periods. However, the pH value decreased during storage, while the titration acidity value increased slightly. This change might be due to the continued metabolic activity of starter cultures and the activity of contaminated yeast and mold during storage [39]. This result exhibited a similarity with Bakr et al. [32] who reported that the growth of all *Lactobacillus bulgaricus* and *Streptococcus thermophilus* could have been due to inulin, which is also found in JA. 

### 2.2. Microbiological Properties of the JA-Supplemented Yogurt Samples

The *Lactobacillus bulgaricus*, *Streptococcus thermophilus* and yeast, and mold counts in the JA-supplemented yogurt samples are presented in Table 2.

Table 2 demonstrates that the addition of 3% JA resulted in an increase in the viable count of *L. bulgaricus*. As presented in Table 2, the highest *L. bulgaricus* count was found in sample JA_3%,_ followed by JA_2%_, JA_1%,_ and the control. Similar results were observed for *S. thermophilus*. The *L. bulgaricus* and *S. thermophilus* counts in all yogurt samples were deemed to be at permisable levels (>7 log cfu/g) for the 21-day cycle of storage, an amount that could provide a health-promoting effect [40]. The statistical evaluations for the control and JA_1%_, and the JA_2%_ and JA_3%_ samples showed similarities between them, while these two groups of yogurts were different (*p* < 0.01) from each other in terms of *L. bulgaricus* counts. However, there were no significant differences among the yogurt samples with respect to *S. thermophilus* counts during the storage period, as supported by the statistical analysis. Similar findings were reported by Bakr et al. [32] in a bioyogurt supplemented with natural sources. Furthermore, El-Kholy and Mahrous [41] reported bioyogurt supplemented with a prebiotic agent obtained from JA. Inulin, vitamins, and organic acids contained in JA tubers are used as a source of energy by the yogurt cultures; the JA-based soluble dietary fibers have a prebiotic effect [42,43,44].

The counts for yeast and mold were not affected by differences in JA concentrations, however, the storage period showed some differences. The storage period had a significant difference on the yeast and mold counts at the level of *p* < 0.01. The microbiological counts revealed that the addition of JA increased bacteria and yeast and mold counts in the supplemented yogurt. The inulin ratio of JA might be responsible for this result [45]. The observed yeast and mold count results using JA and different storage periods may have stemmed from the contamination of these microorganisms during the production.

### 2.3. Organic Acid Evaluations of JA-Added Yogurts

Organic acids are extremely important components for the quality, aroma, and safety of milk and dairy products. During the storage period, nine different organic acids were detected in yogurt samples supplemented with JA (Table 3).

Orotic acid is a natural organic acid that is found in ruminant milk at significant levels. It is formed during the pyrimidine biosynthesis pathway and stimulates the growth of yogurt bacteria. The average amount of orotic acid in milk is between 69 and 74 mg/L, and its amount decreases significantly during the fermentation process [24,46]. As seen in Table 3, the highest mean value (11.43 ± 0.71 μg/g) was measured in the control, while the lowest mean value was determined in JA_3%_ (10.55 ± 0.15 μg/g). Statistical evaluations revealed that the JA_3%_ sample had significantly different acid levels (*p* < 0.01) from other samples.

The orotic acid values of yogurt samples decreased and the last (21st) day of storage revealed a significantly different (*p* < 0.01) level of acid from the other storage days (Table 3). The results also revealed that the orotic acid concentration decreased depending on the JA concentration and the prolonged storage time. Tormo and Izco [25] reported that the orotic acid concentration in fermented milk showed a decrease during the different storage times and fermentation processes.

Pyruvic acid is produced as the substance of carbohydrate and protein metabolism and it is turned into lactic acid and some other metabolites by different enzymes [46,47]. In the yogurt samples, the highest pyruvic acid concentration was observed in the control (6.36 ± 1.12 μg/g), followed by JA_1%_ (6.26 ± 0.71 μg/g), JA_2%_ (6.17 ± 0.84 μg/g), and JA_3%_ (5.51 ± 0.86 μg/g). However, there were no significant differences observed among the samples. 

The pyruvic acid concentrations in the yogurt samples were, however, significantly (*p* < 0.01) decreased on the 21st day of storage. As depicted in Table 3, there were significant differences in the pyruvic acid concentrations during the storage periods (*p* < 0.01). The other observed results in earlier sections might have been caused by the decreasing pyruvic acid concentration in fermented dairy products during storage. This could be caused by the consumption of organic acid by the yogurt starter cultures during the yogurt production process [48].

Citric acid is a natural organic acid found in fruits and juices, plants, and vegetables. It is also present at an average of 0.2% in fresh milk, as a natural component [25,49]. As presented in Table 3, the control exhibited the highest amounts of citric acid (51.52 ± 3.28 μg/g); while the lowest was found in JA_3%_ (44.32 ± 2.06 μg/g). JA_1%_ JA_2%,_ and JA_3%_ exhibited a similar trend and were significantly different from the control (*p* < 0.01). However, storage had no effect on the amount of citric acid, as there were no significant differences observed in the samples. Bhandari and Kawabata [50] reported citric acid as the second most abundant organic acid after succinic acid in wild yam samples.

Lactic acid is the most important organic acid in milk and dairy products. It is produced with lactic acid fermentation by microorganisms. The production of lactic acid is important for the formation of quality characteristics and flavor development in dairy products [24,51]. The control sample had the highest lactic acid value, followed by JA_1%,_ JA_3%,_ and JA_2%_ in that order. The control exhibited a significantly higher level (*p* < 0.01) of the acid from other yogurt samples (Table 3). The lactic acid concentrations in the yogurt samples increased during the 21-day storage and the last day (21st) showed a significantly different amount (*p* < 0.01). The 1st and 7th days of storage were similar, and also 14th and 21st days had a similar trend with respect to statistical evaluations. However, these time-period groups showed differences from each other at the level of *p* < 0.01.

Acetic acid is a secondary product and is formed during the metabolic activity in plants and animals. Acetic acid is produced by microorganisms using lactose, citrate, and amino acids [52]. When acetic acid is formed in excess, it gives a vinegary aroma and an unpleasant taste to yogurt and other fermented kinds of milk [51,53]. The highest mean value of acetic acid (4.78 ± 0.14l μg/g) was found in JA_2%_ followed by JA_1%_, the control, and JA_3%_ (4.30 ± 0.41 μg /g). Additionally, JA_2%_ had statistically significant (*p* < 0.05) differences from the other samples. Furthermore, the acetic acid ratios of yogurt samples showed an increase on the 21st day of storage, and this situation was determined to be statistically significant (*p* < 0.01). It is thought that this increase in acetic acid concentration was due to yeast, mold, and other microorganism activities that continued during storage.

Butyric acid is formed as a result of the breakdown of milk fat and the deamination of amino acids by unwanted microorganisms in milk and its products [54]. The butyric acid concentrations were found between 103.43 ± 29.44 μg/g and 50.61 ± 5.56μg/g for the control and JA_1%_ samples (Table 3). According to the statistical evaluations (Table 3), JA_1%_, JA_2%_, and JA_3%_ were similar, but the control sample was statistically significantly different (*p* < 0.01) from other samples in their butyric acid values. In general, butyric acid values increased during the storage period, but this increase was found to be statistically insignificant.

Propionic acid is an organic acid that occurs frequently in products such as cheese, butter, etc. This substance may be formed as a result of the metabolic activities of animals or plants. Propionic acid can be used to control microbial growth in food products, feeds, cereals, and pharmaceutical products during storage [55]. Table 3 demonstrates that the highest value was found in JA_3%_ and the lowest propionic acid concentration was found in JA_2%_. The JA_3%_ sample was statistically different from other samples at the level of *p* < 0.01. In addition, the 21st day of storage showed a statistically significant difference from the other storage periods. Similar results were reported by Akalın et al. [56]. They also reported that the amount of propionic acid increased during yogurt-making and fermentation process storage.

Malic acid is formed as a result of the metabolic activity of plants and animals. This organic acid is generally used in food products as a flavor enhancer and acidity-increasing agent [57]. The highest malic acid value was in JA_3%_ (39.53 ± 1.27 μg/g) and it was followed by JA_2%_ (39.31 ± 1.68 μg/g), JA_1%_ (37.78 ± 0.66 μg/g), and the control (0.00 ± 0.00 μg/g), respectively (Table 3). The JA_2%_ and JA_3%_ were similar to each other, but other yogurts demonstrated statistically important differences (*p* <0.01) from each other and these two yogurt samples. On the other hand, the effect of storage on malic acid concentration was found to be insignificant. Malic acid is a natural organic acid of fruits and plants. Generally, it is not found in milk and products naturally. Malic acid detected in yogurt samples in our study was due to the JA added to yogurts. The Jerusalem artichoke contains a large number of organic polyacids, including citric acid, malic acid, raspberry acid, succinic acid, and fumaric acid [58]. However, a low amount of malic acid was determined in the samples due to the low amount of JA added to the yogurts.

Succinic acid is produced by some *Lactobacillus* species. This acid has an acidic, bitter, salty taste. When it occurs in dairy products in excess, it gives an undesirable taste and aroma to the product [55]. Table 3 shows that the highest mean value was found in the control and the lowest value was found in JA_1%_. Additionally, the control sample was statistically different from other samples at the level of *p* < 0.01. However, storage periods did not affect the succinic acid ratios of samples. Bhandari and Kawabata [50] found that succinic acid ranged between 119 and 2510 mg/100 g in four species of wild yam samples. Furthermore, succinic acid was found as a prominent organic acid for the yogurt cultures.

### 2.4. Sensory Evaluations of the JA Added Yogurts

Sensory scores of the JA-added yogurts were given on a rating scale from one (very poor) to five (excellent), as presented in Table 4.

The addition of JA to the yogurts in different ratios and storage periods affected the scores of appearance and consistency at the level of *p* < 0.01, but odor scores were affected statistically by the JA ratio and 21 days of storage at the level of *p* < 0.05 level. However, the flavor scores of panelists were not affected by the addition of JA and storage periods. Table 4 shows the highest appearance and floor scores were given to JA_3%_ by the sensory panelists. Additionally, the control had the highest consistency scores and JA_1%_ was evaluated as the best by the panelists. When the effect of the storage period on sensory parameters was examined, it was determined that all of them decreased during the 21-day storage period. Santis and Frangipane [59] reported that some important sensory properties of JA allowed it to be consumed both raw and cooked. Generally, the addition of JA at 1, 2, and 3% affected the acceptance of the yogurts. Briefly, the preference of taste positively affected the overall acceptability of supplemented yogurts with JA tubers. However, the flavor, appearance, consistency, odor, and flavor score of yogurt samples gradually decreased during storage.

## 3. Materials and Methods

### 3.1. Materials

Fresh cow’s milk (25L) and nonfat powdered milk (used in yogurt production for standardization) were purchased from commercial sources (Enka Dairy Products) in Konya, Türkiye. The JA tubers were collected in October 2018 from Erzurum, Türkiye. Commercial freeze-dried yogurt thermophilic lactic cultures (YoFlex^®^M780) including *Streptococcus thermophilus* and *Lactobacillus delbrueckii* ssp. *bulgaricus* were obtained from Chr. Hansen, Istanbul, Türkiye. Technological properties of the commercial cultures from yogurts were investigated and validated by Asensio-Vegas et al. [60]. Additionally, the yogurt-starter bacteria strains were isolated from original homemade-prepared yogurt by the producer in Türkiye (Peyma Chr. Hansen). These strains were isolated from the commercial freeze-dried cultures that were confirmed by API 50 CH (bioMérieux, France) and partial 16S rDNA sequencing.

### 3.2. Preparation of Jerusalem Artichoke

Tubers of JA were washed in tap water and any degraded pieces were removed before being sliced. To inhibit polyphenol oxidase activity, the sliced tubers were soaked in boiling water for 5 min, then immediately dipped in (1%) cold citric acid solution [38]. The tuber slices were crushed using a blender (Tefal Blender). This method was used with the aim of eliminating the bitterness of JA tubers.

### 3.3. The Production of JA-Supplemented Yogurt Samples

The cows’ milk was heated to 85–90 °C for 10–15 min and then cooled to 42–43 °C. The milk was divided into four parts. Milk bases were inoculated with a direct vat set lyophilized starter culture in the ratio (3 g/100 mL) suggested by the supplier. The inoculated milk was incubated at 42 ± 1 °C until the pH reached 4.6. After, obtained yogurts were held in a refrigerator at 4 ± 1 °C for 24 h. The yogurt without supplementation was used as control while treatments were supplemented with 1, 2, and 3% of JA tuber samples and represented as JA_1%_, JA_2%,_ and JA_3%_ respectively. The yogurt samples were transferred to 150 mL sterile plastic containers and stored at 4 °C for 21 days of storage. The analyses were completed at 1, 7, 14, and 21 days. Yogurt samples were prepared with milk taken at two different times and in two replications at different times.

### 3.4. Chemical Analysis of JA-Added Yogurts

The total solids and ash contents of yogurt samples were measured with gravimetric methods and ash by heating a 5 g sample in a muffle furnace at 100 °C for 1 h, 200 °C for 2 h, and 550 °C overnight. Fat content was determined by the Gerber method and protein ratio by the Kjeldahl method [61]. The titration acidity value of yogurt samples was found with a titration method using phenolphthalein as an indicator and a 0.1 N NaOH solution. The pH was determined by a pH meter (model WTW pH-340-A, Weilheim, Germany) at room temperature. Syneresis ratios of yogurts were measured with the method of Dönmez et al. [62]. Briefly, the syneresis rates of yogurts were determined by a centrifugal acceleration test. Five grams of yogurt sample were placed in a test tube and centrifuged at 1200× *g* for 0, 3, 6, 9, 12, and 15 min at room temperature. At each time interval, the volume of the serum separated from the samples was measured to estimate the initial rate of syneresis, which was expressed as milliliters of serum released per gram of sample per unit of time. The viscosity of yogurts was determined with a digital rotational viscometer (DV-II + Pro, Brookfield Engineering Laboratories Inc., Stoughton, MA, USA) with a spindle No.64. The speed of the spindle was adjusted to 50 rpm. The viscosity values of yogurts were expressed as centipoises (cP) [63].

### 3.5. Microbiological Analysis of JA-Supplemented Yogurt Samples

The *Lactobacillus bulgaricus* count was completed using MRS agar (Oxoid Ltd., Basingstoke, Hampshire, UK) which was incubated anaerobically at 37 °C for 72 h. M17 agar (Oxoid Ltd., Basingstoke, Hampshire, UK) was used for the determination of *S. thermophilus* and incubated in the aerobic environment at 37 °C for 24–48 h. Mould and yeasts were enumerated on DRBC (Dichloran Rose–Bengal Chloramphenicol Agar, Oxoid) agar that included 0.002% Dichloran and 0.025% Rose–Bengal with incubation at 25 °C for 5 days [51,64].

### 3.6. Organic Acid Analysis of JA-Supplemented Yogurt Samples

The organic acid profiles of experimental yogurt samples were performed using Agilent HPLC 1100 series G 1322 A, Waldbronn, Germany according to the method of Fernandez-Garcia and McGregor [24] and Kavaz and Bakırcı [51]. Briefly, 4 g of yogurt was mixed with 25 mL of 0.001 N H_2_SO_4_ and then centrifuged at 5000× *g* for 10 min. The obtained supernatants were filtered using Whatman No.1 filter papers and a 0.45 μM membrane filter (PALL, Ann Arbor, MI, USA). The aliquots were transferred to HPLC vials and stored at −20 °C until analysis. For this analysis, 0.001N H_2_SO_4_ was used as a mobile phase for a 0.6 mL/ min flow rate at 210 nm. Organic acids were separated using a Alltech IOA-1000 organic acid column (300 × 7.8 mm, Alltech, IL, USA). For each sample, duplicate injections (approximately 10 μL) were performed [31].

### 3.7. Sensory Analysis of JA-Supplemented Yogurt Samples

The sensorial scoring of yogurt samples was determined by a panel of 15 trained panelists (aged 25–55 years, seven females and eight males) in a dedicated sensory evaluation laboratory. Panelists were selected and trained according to UNE 87024:1-95 and ISO/DIS 22935-IDF 99. Appearance, consistency, odor, and flavor properties of the yogurts were determined on a scale of 1–5 (1: poor, to 5: excellent) on days 1, 7, 14, and 21 of storage. The yogurts were given to panelists in plastic sterile cups. Water and bread were served to the panelists to clean their mouths between the samples [60,65]. All the sensory analyses were carried out on samples at 13 ± 2 °C. Overall acceptability was calculated from the total score of the judged attributes [38].

### 3.8. Statistical Analysis of JA-Supplemented Yogurt Samples

The results were analyzed using the SPSS version 28 (SPSS Inc., Chicago, IL, USA) statistical software. Analyses of variance (ANOVA) of Duncan’s multiple range tests were used to evaluate differences in terms of the mean values. All measurements were performed in duplicate.

## 4. Conclusions

This study revealed that the addition of JA to yogurt at various concentrations improved the functional characteristics of yogurt. The JA tubers can be considered an important prebiotic agent in yogurt production due to their inulin content, as well as an important product in terms of their positive effects on health. The results showed that JA improved the physicochemical, rheological, and sensorial properties of the yogurt and maintained the viability of the yogurt cultures. It is also of great importance in terms of providing the opportunity to produce a novel and functional product according to consumer demands. Briefly, consumers prefer natural and functional products at a high level. In conclusion, it can be said that JA is an important additive for yogurt production due to its characteristic properties.

## Figures and Tables

**Table 1:** The effect of JA ratios and storage periods on the physicochemical properties of the experimental yogurts (mean ± SD).

Experimental Yogurts		Total Solids (%)	Ash (%)	Protein (%)	Fat (%)	Apparent Viscosity (cP)^b^ (50 rpm)	Syneresis (mL/25 g)	Titratable Acidity (%)	pH
Control (C)		13.56 ± 0.38a *	0.64 ± 0.02a **	4.12 ± 0.58a **	3.44 ± 0.12b **	6950 ± 504.27	5.85 ± 0.37c **	0.84 ± 0.07a **	4.54 ± 0.09ab *
JA_1%_		14.25 ± 0.25b *	0.75 ± 0.03b **	5.08 ± 0.56b **	3.39 ± 0.12b **	7256 ± 364.92	5.36 ± 0.39b **	0.82 ± 0.08a **	4.45 ± 0.08a *
JA_2%_		14.19 ± 0.26b *	0.76 ± 0.03b **	5.15 ± 0.38b **	3.44 ± 0.07b **	7348 ± 471.35	4.83 ± 0.62a **	0.92 ± 0.05b **	4.43 ± 0.14a *
JA_3%_		14.14 ± 0.65b *	0.74 ± 0.04b **	5.26 ± 0.46b **	3.21 ± 0.16a **	7400 ± 423.42	4.62 ± 0.40a **	1.24 ± 0.07c **	4.40 ± 0.12b *
**Storage time (days)**									
1	(C)	13.98 ± 0.52a *	0.71 ± 0.06a **	4.63 ± 0.74a **	3.32 ± 0.08a **	7138 ± 303.26	5.43 ± 0.77c **	0.92 ± 0.16a **	4.55 ± 0.13ab *
	JA_1%_	14.32 ± 0,22b *	0.75 ± 0.03b **	5.10 ± 0.12b **	3.48 ± 0.06b **	7182 ± 372.28	5.33 ± 0.23b **	1.12 ± 0.08a **	4.44 ± 0.08a *
	JA_2%_	14.56 ± 0,32b *	0.78 ± 0.05b **	5.16 ± 0.16b **	3.52 ± 0.04b **	7398 ± 452.32	4.78 ± 0.24a **	1.18 ± 0.10b **	4.40 ± 0.12a *
	JA_3%_	14.98 ± 0,28b *	0.80 ± 0.04b **	5.31 ± 0.22b **	3.54 ± 0.08a **	7466 ± 412.22	4.56 ± 0.28a **	1.26 ± 0.09c **	4.38 ± 0.08b *
7	(C)	14.02 ± 0.22a *	0.72 ± 0.06a **	4.85 ± 0.61a **	3.29 ± 0.16a **	7213 ± 434.86	5.13 ± 0.40c **	0.95 ± 0.18a **	4.49 ± 0.07ab *
	JA_1%_	14.28 ± 0.32b *	0.78 ± 0.04b **	5.02 ± 0.32b **	3.44 ± 0.17b **	7456 ± 396.88	5.02 ± 0.11b **	1.21 ± 0.06a **	4.39 ± 0.06a *
	JA_2%_	14.66 ± 0.25b *	0.81 ± 0.03b **	5.18 ± 0.38b **	3.56 ± 0.12b **	7.502 ± 324.10	4.66 ± 0.28a **	1.28 ± 0.07b **	4.33 ± 0.14a *
	JA_3%_	15.02 ± 0.21b *	0.86 ± 0.08b **	5.54 ± 0.20b **	3.62 ± 0.22b **	7.586 ± 286.22	4.48 ± 0.16a **	1.31 ± 0.04c **	4.29 ± 0.09b *
14	(C)	13.92 ± 0.48a *	0.72 ± 0.06a **	5.04 ± 0.70a **	3.44 ± 0.15a **	7261 ± 590.56	5.10 ± 0.69c **	0.95 ± 0.20a **	4.40 ± 0.03ab *
	JA_1%_	14.12 ± 0.12b *	0.76 ± 0.04b **	5.18 ± 0.52b **	3.54 ± 0.12b **	7298 ± 458.25	4.88 ± 0.72b **	1.06 ± 0.02a **	4.35 ± 0.09a *
	JA_2%_	14.36 ± 0.28b *	0.84 ± 0.06b **	5.66 ± 0.42b **	3.58 ± 0.08b **	7402 ± 365.23	4.54 ± 0.32a **	1.19 ± 0.08b **	4.31 ± 0.06a *
	JA_3%_	14.98 ± 0.12b *	0.87 ± 0.05b **	5.71 ± 0.22b **	3.64 ± 0.24b **	7566 ± 298.28	4.28 ± 0.12a **	1.29 ± 0.06c **	4.28 ± 0.08b *
21	(C)	14.13 ± 0.55a *	0.74 ± 0.05a **	5.08 ± 0.63a **	3.44 ± 0.15a **	7344 ± 521.97	5.01 ± 0.72c **	1.00 ± 0.21a **	4.39 ± 0.10ab *
	JA_1%_	14.39 ± 0.27b *	0.83 ± 0.02b **	5.22 ± 0.21b **	3.49 ± 0.11b **	7442 ± 421.12	4.88 ± 0.12b **	1.08 ± 0.09a **	4.32 ± 0.12a *
	JA_2%_	14.74 ± 0.22b *	0.88 ± 0.03b **	5.47 ± 0.33b **	3.55 ± 0.14b **	7588 ± 326.21	4.62 ± 0.48a **	1.19 ± 0.05b **	4.29 ± 0.08a *
	JA_3%_	15.06 ± 0.02b *	0.91 ± 0.01b **	5.68 ± 0.12b **	3.66 ± 0.12b **	7.654 ± 258.22	4.48 ± 0.12a **	1.31 ± 0.03c **	4.24 ± 0.12b *

Mean values ± standard deviations of yogurt manufacturing with duplicate samples. The letters a, b, c, and d indicate means that are significantly different at *p* < 0.01 and *p* < 0.05 levels **: *p* < 0.01, *: *p* < 0.05.

**Table 2:** The effect of JA ratios and storage periods on physicochemical properties of the experimental yogurts (mean ± SD).

Experimental Yogurts		*Lactobacillus Bulgaricus* Count (log cfu/g)	*Streptococcus Thermophilus* Count (log cfu/g)	Yeast and Mold Count (log cfu/g)
Control		7.32 ± 0.28a **	7.63 ± 0.42	1.91 ± 1.82
JA_1%_		7.39 ± 0.23a **	7.86 ± 0.42	2.03 ± 1.76
JA_2%_		7.61 ± 0.15b	7.93 ± 0.24	1.78 ± 1.19
JA_3%_		7.70 ± 0.06b **	7.93 ± 0.48	2.24 ± 1.98
**Storage time (days)**				
1	Control	7.47 ± 0.23a **	7.65 ± 0.44	1.22 ± 1.88ab **
	JA_1%_	7.54 ± 0.25a **	7.88 ± 0.24	1.36 ± 1.20
	JA_2%_	7.73 ± 0.15b **	7.92 ± 0.12	1.32 ± 1.10
	JA_3%_	7.81 ± 0.18b **	7.98 ± 0.11	1.82 ± 1.24
7	Control	7.58 ± 0.23a **	7.91 ± 0.32	0.83 ± 0.97a **
	JA_1%_	7.72 ± 0.21a **	7.96 ± 0.22	0.89 ± 0.27
	JA_2%_	7.79 ± 0.12b **	8.02 ± 0.12	0.95 ± 0.52
	JA_3%_	7.83 ± 0.13b **	8.08 ± 0.10	1.12 ± 0.88
14	Control	7.39 ± 0.29a **	7.73 ± 0.37	2.62 ± 1.10bc **
	JA_1%_	7.56 ± 0.21a	7.85 ± 0.21	2.65 ± 1.12
	JA_2%_	7.65 ± 0.14b **	7.89 ± 0.11	2.71 ± 1.54
	JA_3%_	7.71 ± 0.15b **	8.02 ± 0.12	2.88 ± 1.68
21	Control	7.58 ± 0.22a **	8.06 ± 0.40	3.31 ± 1.24c **
	JA_1%_	7.66 ± 0.14a **	8.10 ± 0.20	3.38 ± 1.32
	JA_2%_	7.74 ± 0.2b **	8.12 ± 0.12	3.44 ± 1.30
	JA_3%_	7.88 ± 0.16b **	8.15 ± 0.22	3.51 ± 1.44

Mean values ± standard deviations yogurts manufacturing with duplicate samples. The letters a, b, c, and d indicate means that were significantly different at *p* < 0.01 and *p* < 0.05 levels **: *p* < 0.01, *: *p* < 0.05.

**Table 3:** Organic acid profiles of JA concentrations and experimental yogurts during different storage periods (mean ± SD).

Yogurts		Orotic Acid (μg/g)	Pyruvic Acid (μg/g)	Citric Acid (μg/g)	Lactic Acid (μg/g)	Acetic Acid (μg/g)	Butyric Acid (μg/g)	Propionic Acid (μg/g)	Malic Acid (μg/g)	Succinic Acid (μg/g)
Control		11.43 ± 0.71 **	6.36 ± 1.12	51.52 ± 3.28 **	1208.66 ± 62.43 **	4.37 ± 0.45a *	103.43 ± 29.4 **	1.88 ± 0.52a *	0.00 ± 0.00a **	4.75 ± 1.25 *
JA_1%_		11.20 ± 0.51b *	6.26 ± 0.71	46.10 ± 1.64a *	1084.67 ± 45.65a **	4.47 ± 0.36a *	50.61 ± 5.56a **	1.81 ± 0.46a *	37.78 ± 0.66b *	2.38 ± 0.52a **
JA_2%_		11.09 ± 0.33b **	6.17 ± 0.84	46.35 ± 0.79a **	1066.04 ± 37.98a **	4.78 ± 0.14b *	53.98 ± 5.59a **	1.75 ± 0.38a **	39.31 ± 1.68c **	2.63 ± 0.44a **
JA_3%_		10.55 ± 0.15a **	5.51 ± 0.86	44.32 ± 2.06a **	1068.02 ± 58.32a **	4.30 ± 0.41a *	60.10 ± 10.26a **	2.94 ± 0.98b **	39.53 ± 1.27c **	3.06 ± 0.50a **
**Storage time(days)**										
1	Control	11.42 ± 0.61b **	6.92 ± 0.60c **	46.49 ± 3.18	1036.31 ± 32.36a **	4.24 ± 0.53a **	62.70 ± 25.51	1.62 ± 0.69a **	0.00 ± 0.00a **	3.13 ± 1.73
	JA_1%_	11.22 ± 0.21b *	6.83 ± 0.64	40.80 ± 1.12	1002.24 ± 28.32	4.32 ± 0.41	64.41 ± 19.21	1.59 ± 0.32	40.12 ± 0.72	2.14 ± 0.15
	JA_2%_	11.10 ± 0.32b **	6.72 ± 0.72	41.01 ± 0.80	1009.12 ± 42.50	4.48 ± 0.12	66.12 ± 15.12	1.52 ± 0.22	41.12 ± 0.54	2.36 ± 0.21
	JA_3%_	10.58 ± 0.18a **	6.12 ± 0.56	39.2 ± 1.32	1011.04 ± 36.24	4.12 ± 0.18	68.24 ± 12.39	2.12 ± 0.72	41.88 ± 0.11	2.96 ± 0.18
7	Control	11.17 ± 0.46b **	6.35 ± 0.36bc **	46.85 ± 2.13	1064.11 ± 33.90a **	4.32 ± 0.20a **	65.06 ± 19.80	2.06 ± 0.62a **	0.00 ± 0.00a **	2.69 ± 0.84
	JA_1%_	11.02 ± 0.32	6.24 ± 0.24	41.02 ± 1.72	1006.06 ± 22.50	4.48 ± 0.44	63.02 ± 12.24	1.99 ± 0.24	42.55 ± 0.51	1.98 ± 0.16
	JA_2%_	10.88 ± 0.12	6.14 ± 0.18	43.10 ± 0.92	1011.12 ± 28.00	4.63 ± 0.21	66.80 ± 14.56	1.90 ± 0.11	43.28 ± 0.31	2.24 ± 0.32
	JA_3%_	10.32 ± 0.20	5.96 ± 0.12	40.12 ± 1.02	1015.10 ± 28.20	4.32 ± 0.28	69.12 ± 12.20	2.32 ± 0.72	43.10 ± 0.58	2.88 ± 0.24
14	Control	11.06 ± 0.54b **	5.57 ± 0.89ab **	48.25 ± 5.03	1151.58 ± 29.58b **	4.52 ± 0.27a **	69.72 ± 28.78	2.00 ± 0.38a **	0.00 ± 0.00a **	3.44 ± 0.86
	JA_1%_	10.98 ± 0.30	5.29 ± 0.22	42.98 ± 1.52	1072.54 ± 22.25	4.65 ± 0.32	72.12 ± 14.32	1.92 ± 0.24	44.32 ± 0.24	2.08 ± 0.54
	JA_2%_	10.74 ± 0.26	5.20 ± 0.16	44.20 ± 1.20	1091.21 ± 32.24	4.78 ± 0.38	75.05 ± 21.12	1.88 ± 0.54	45.56 ± 0.72	2.22 ± 0.14
	JA_3%_	10.21 ± 0.12	5.02 ± 0.20	41.12 ± 1.12	1101.10 ± 24.30	4.50 ± 0.25	78.24 ± 16.18	2.24 ± 0.22	46.02 ± 0.42	2.96 ± 0.10
21	Control	10.62 ± 0.33a **	5.47 ± 0.91a **	46.70 ± 3.03	1175.38 ± 40.41b **	4.84 ± 0.21b **	78.43 ± 31.57	2.69 ± 1.00b **	0.00 ± 0.00a **	3.56 ± 1.12
	JA_1%_	10.12 ± 0.18	5.20 ± 0.45	43.15 ± 1.64	1085.22 ± 20.24	5.01 ± 0.45	80.24 ± 18.00	2.56 ± 0.48	45.10 ± 0.36	2.10 ± 0.10
	JA_2%_	10.01 ± 0.15	5.11 ± 0.18	44.36 ± 1.24	1094.26 ± 18.25	5.18 ± 0.32	82.80 ± 16.12	2.38 ± 0.42	47.11 ± 0.33	2.25 ± 0.15
	JA_3%_	10.00 ± 0.10	4.92 ± 0.10	40.90 ± 1.10	1116.30 ± 32.20	4.96 ± 0.52	84.20 ± 14.58	2.88 ± 0.62	48.02 ± 0.48	3.01 ± 0.10

Mean values ± standard deviations yogurts manufacturing with duplicate samples. The letters a, b, c, and d indicate means that are significantly different at *p* < 0.01 and *p* < 0.05 levels **: *p* < 0.01, *: *p* < 0.05.

**Table 4:** The sensory evaluation of experimental yogurts depends on JA ratios and storage times (mean ± SD).

Experimental Yogurts		Appearance	Consistency	Odor	Flavor
Control		4.41 ± 0.31bc **	4.49 ± 0.26b **	4.38 ± 0.28b *	4.11 ± 0.32b *
JA_1%_		4.11 ± 0.32a **	4.39 ± 0.32b **	4.40 ± 0.35a *	4.03 ± 0.23a *
JA_2%_		4.21 ± 0.38ab **	4.10 ± 0.16a **	4.24 ± 0.27bc *	4.07 ± 0.23a *
JA_3%_		4.55 ± 0.39c **	4.31 ± 0.21b **	4.39 ± 0.18b *	4.26 ± 0.26c *
**Storage time (days)**					
1	Control	4.50 ± 0.27b **	4.55 ± 0.24b **	4.44 ± 0.18b *	4.14 ± 0.30b *
	JA_1%_	4.20 ± 0.18a **	4.41 ± 0.22b **	4.29 ± 0.12a *	4.10 ± 0.10a *
	JA_2%_	4.32 ± 0.24ab **	4.18 ± 0.32a **	4.26 ± 0.10bc *	4.22 ± 0.12a *
	JA_3%_	4.62 ± 0.34c **	4.42 ± 0.18b **	4.38 ± 0.14b *	4.52 ± 0.20c *
7	Control	4.58 ± 0.29b **	4.39 ± 0.27b **	4.26 ± 0.30ab *	4.21 ± 0.21b *
	JA_1%_	4.32 ± 0.14a **	4.12 ± 0.21b **	4.21 ± 0.10a *	4.15 ± 0.10a *
	JA_2%_	4.43 ± 0.13ab **	4.30 ± 0.16a **	4.35 ± 0.18bc *	4.18 ± 0.10a *
	JA_3%_	4.52 ± 0.16c **	4.48 ± 0.12b **	4.56 ± 0.24b *	4.32 ± 0.18c *
14	Control	4.16 ± 0.21a **	4.18 ± 0.18a **	4.20 ± 0.35ab *	4.17 ± 0.23b *
	JA_1%_	4.01 ± 0.10b **	4.10 ± 0.12a **	4.12 ± 0.16a *	4.10 ± 0.12a *
	JA_2%_	4.10 ± 0.14b **	4.24 ± 0.22a **	4.30 ± 0.12bc *	4.18 ± 0.10a *
	JA_3%_	4.45 ± 0.16c **	4.36 ± 0.18b **	4.46 ± 0.18b *	4.28 ± 0.26c *
21	Control	4.04 ± 0.45a **	4.18 ± 0.24a **	4.11 ± 0.33a *	3.96 ± 0.30b *
	JA_1%_	4.00 ± 0.01a **	4.12 ± 0.16a **	4.16 ± 0.18a *	4.02 ± 0,20a *
	JA_2%_	4.12 ± 0.08b **	4.01 ± 0.08a **	4.28 ± 0.24bc *	4.10 ± 0.12a *
	JA_3%_	4.26 ± 0.10c **	4.14 ± 0.12b **	4.40 ± 0.18b *	4.28 ± 0.22c *

Mean values ± standard deviations of yogurt manufacturing with duplicate samples. The letters a, b, c, and d indicate means that are significantly different at *p* < 0.01 and *p* < 0.05 levels **: *p* < 0.01, *: *p* < 0.05.

## Data Availability

Not applicable.
